# An entry-competent intermediate state of the HIV-1 envelope glycoproteins

**DOI:** 10.14800/rci.1544

**Published:** 2017-05-24

**Authors:** Alon Herschhorn, Joseph Sodroski

**Affiliations:** 1 Department of Cancer Immunology and Virology, Dana-Farber Cancer Institute, Boston, 02215, MA, USA; 2 Department of Microbiology and Immunobiology, Harvard Medical School, Boston, 02215, MA, USA; 3 Department of Immunology and Infectious Diseases, Harvard T. H. Chan School of Public Health, Boston, 02215, MA, USA

**Keywords:** HIV-1, envelope glycoproteins, intermediate states, conformation

## Abstract

The human immunodeficiency virus type 1 (HIV-1) envelope glycoproteins (Env) mediate viral entry and are the sole target of neutralizing antibodies. Recent studies show that the metastable HIV-1 Env trimer can transit among three conformational states: State 1, State 3, and State 2, corresponding to the “closed”, “open” and intermediate conformations, respectively. During virus entry, binding to the CD4 receptor drives Env from state 1 to state 3. In the unliganded Env, transitions from the closed (State 1) conformation are restrained by intramolecular interactions among different Env residues, which regulate HIV-1 Env conformation. Releasing the specific restraints on State 1 Env leads to increased occupancy of State 2, which is a functional conformation on the entry pathway and an obligate intermediate between State 1 and State 3. Frequent sampling of intermediate State 2 allows HIV-1 to infect cells expressing low levels of CD4, and leads to resistance to several broadly neutralizing antibodies as well as small-molecule inhibitors. Recent findings provide new mechanistic insights into the function and inhibition of HIV-1 Env and will contribute to the development of new therapeutic and prophylactic interventions to combat HIV-1.

## The human immunodeficiency virus type-1 envelope glycoproteins

Approximately 36.7 million people are infected with the human immunodeficiency virus type I (HIV-1) worldwide (www.who.int). Current antiretroviral treatment is effective and reduces viremia to undetectable levels in most patients, significantly decreasing the mortality and morbidity of infected individuals. Nevertheless, the acquired immunodeficiency syndrome (AIDS) epidemic is stably sustained by ∼2 million new infections each year, mainly because a curative treatment and/or an effective vaccine for HIV-1 prevention are not yet available. New approaches are currently being explored to allow detailed understanding of the latent reservoir of HIV-1 in infected individuals ^[1]^ to develop broadly neutralizing antibodies as preventive and therapeutic modalities ^[2]^; and to devise novel approaches to address HIV-1 persistence and allow long-term control of the virus without the need for antiretroviral drugs ^[3]^.

HIV-1 entry is mediated by the interaction of the HIV-1 envelope glycoproteins (Env) with the CD4 receptor and CCR5/CXCR4 coreceptor. Three gp120 exterior subunits are noncovalently associated with three gp41 transmembrane subunits to form the HIV-1 Env trimer ^[4, 5]^, and there are approximately 10-14 trimeric spikes on each HIV-1 virion. The low number of spikes and Env conformational dynamics are important for the maintenance of a delicate balance between the requirements to interact with host receptors and the necessity to avoid neutralizing antibodies. Each subunit is associated with specific activity: the gp120 subunit recognizes the host receptors and gp41 facilitates membrane fusion. Binding of gp120 to the CD4 receptor induces the transition of Env from a metastable, high-potential energy state to downstream conformations. CD4-induced Env transitions lead to extensive structural rearrangements that include a repositioning of the V1/V2 and V3 loops, formation of the bridging sheet and coreceptor binding site, and formation/exposure of gp41 heptad repeat (HR1) coiled coil ^[6-18]^. Subsequent binding to the CCR5 or CXCR4 coreceptor promotes the formation of a stable gp41 six-helix bundle, composed of the HR1 and HR2 heptad repeats, a process that is thought to drive the fusion of the viral and host cell membranes ^[19-23]^.

## Conformational transitions of HIV-1 Env

Structural studies of the HIV-1 Env trimer on the surface of virions revealed that the unliganded Env trimer adopts a closed conformation, in which the variable loops protect the internal regions from the immune system and premature activation ^[24]^. Numerous reports have documented the ability of amino acid changes in different Env domains to alter Env sensitivity to cold, antibodies and entry inhibitors ^[25-30]^. These amino acid changes affect the propensity of the Env to sample downstream conformations, a property termed intrinsic reactivity ^[27]^. These observations support the concept that the native, unliganded Env trimer of primary HIV-1 strains is metastable and only infrequently samples downstream conformations ^[31]^.

**Figure 1:**
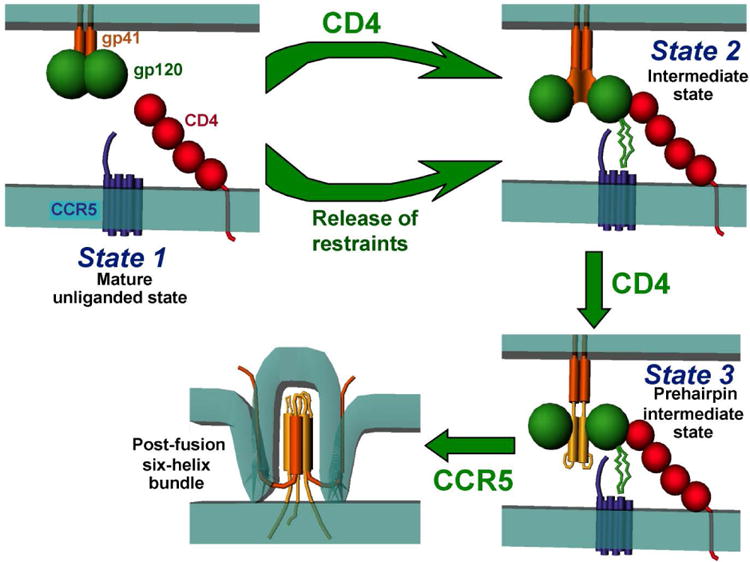
HIV-1 Env conformational states relevant to virus entry The unliganded mature HIV-1 Env trimer is primarily in State 1, a “closed” conformation resistant to neutralization by antibodies. Binding to the receptor, CD4, drives Env into State 2, an intermediate, partially open conformation. Env can also transit into State 2 by modification of gp120 or gp41 residues that restrain the Env trimer in State 1. These State 2 viruses can use low levels of CD4 to enter cells, but exhibit increased sensitivity to antibody neutralization. Additional binding of CD4 to the State 2 Env forms the prehairpin intermediate, an “open” conformation that can bind the second receptor, CCR5. CCR5 binding is thought to promote the formation of a stable six-helix bundle in gp41, driving the fusion of the viral and target cell membranes.

Recent biophysical and biochemical studies now lay a new groundwork for understanding the function and inhibition of HIV-1 Env ^[32, 33]^. The HIV-1 Env trimer, either unliganded or in response to CD4 binding, transits between three states: State 1, State 2, and State 3 (Figure 1). The Env of primary isolates like HIV-1_JR-FL_ predominantly occupies the “closed” State 1 conformation. State 3 represents the CD4-bound conformation and is significantly stabilized by incubation of the Env with soluble CD4 and 17b, an antibody that recognizes the coreceptor binding site ^[33]^. The identity and functional significance of State 2, which resulted in a high-FRET signal in single-molecule fluorescence resonance energy transfer (smFRET) studies, was initially unknown. Later studies identified State 2 as a functional intermediate by linking the increased occupancy of State 2 with hypersensitivity to various ligands that recognize downstream conformations ^[32]^. In particular, hydrophilic changes in Leucine 193 in the V1/V2 loop, which forms the trimer apex, resulted in the release of restraints that maintain a State 1 Env conformation and increased the occupancy of State 2. Further analysis of the trajectory between State 1 and State 3 revealed that all transitions must occur through the intermediate State 2. The results allowed a description of the energy landscape of HIV-1 Env and showed that both CD4 binding and changes in control points that release State-1-restraints lower the activation energy between State 1 and downstream conformations. However, there may be some differences in how the two stimuli induce downstream conformations of Env. The release of State-1-restaints is associated with multiple different Env residue changes, suggesting that the effect of these changes is mainly due to destabilizing State 1. CD4 binding may also destabilize State 1 to some extent. The high degree of enthalpy and entropy change associated with CD4 binding suggests that, in addition, a State 3 Env conformation is stabilized by the interaction with CD4. Integration of these concepts allows an improved understanding of HIV-1 Env function. The Env trimer is metastable and contains several control points that maintain a State 1 conformation; changing these points or binding to the CD4 receptor shifts the distribution of Env conformations among the three defined states (State 1, State 2, and State 3) and increases the occupancy of downstream conformations (State 2 and State 3). This process takes advantage of the natural tendency of the unrestrained gp120 core to assume the CD4-bound (State 3) conformation ^[31]^.

## Conformational dynamics of HIV-1 Env allow adaptability to different host environments

The ability of HIV-1 Env to transit to State 2 provided new insights into the biology of infection of different target cells under physiological conditions. Frequent sampling of State 2 allows more efficient infection of cells that express low levels of the CD4 receptor on their surface. Such a property is a significant advantage for infection of primary macrophages and CD4+ T cells that express low levels of CD4. However, because “open” or partially “open” Env conformations are susceptible to neutralization by many antibodies present in the serum of HIV-1-infected individuals, it is likely that viral tropism (towards macrophages and low-level CD4^+^ T cells) is further shaped by additional physiological constraints *in vivo*.

The ability to manipulate the conformational state of HIV-1 Env provided new opportunities to define the selectivity of broadly neutralizing antibodies (bNAbs). These antibodies are elicited in a minority of HIV-1-infected individuals after a long period of infection, and are considered central to vaccine development. In general, this analysis indicated that bNAb selectivity is associated with specific Env epitopes. The bNAbs targeting the gp120 CD4-binding site and the quaternary V1/V2 loop exhibit preferences for State 1 Env. In contrast, anti-gp41 antibodies that target the membrane proximal external region show a high preference for downstream conformations (State 2 and State 3). These observations will provide further guidance to future design of new immunogens.

Variation in or near several gp120 restraining residues can influence the propensity of HIV-1 Env to sample downstream conformations. Envs from natural HIV-1 isolates exhibit a continuum of Env reactivities ^[27, 34]^. Recognition of State 2 as a functional intermediate raises the possibility that these phenotypes of primary HIV-1 variants are determined by differences in the Env energy landscape. We suggest that variation in Env reactivity among primary HIV-1 viruses potentially reflects altered activation barriers between State 1 and State 2, modulating the degree of sampling of State 2. For viruses with some increase in Env reactivity, the consequent disadvantage of increased susceptibility to neutralization by commonly elicited antibodies may be balanced by the ability to evade State 1-recognizing antibodies like the CD4-binding site and quaternary V1/V2 bNAbs and to trigger the entry process with lower levels of target cell CD4. Thus, variation in the propensity to sample downstream states in different HIV-1 strains augments the ability of the virus to adapt to different target cells and to avoid humoral immune responses.

## Future research

The identification of different Env residue changes that result in varied propensity to sample downstream conformations raises the possibility that additional substates may exist. Indeed, we expect that new advances in smFRET technology, which is used to study the conformational dynamics of HIV-1 Env, will allow high-resolution analysis and facilitate the detection and mapping of substates on the entry pathway of HIV-1. Advanced studies may link the energetic profile of different primary isolates to their infectivity and neutralization properties. The identification of restraining residues that maintain State 1 provides new tools to study the regulation of HIV-1 function and to trap the Env in intermediate states for structural studies. Understanding the molecular mechanism of HIV-1 Env function will provide new approaches to design immunogens and expedite the development of an effective HIV-1 vaccine.

## Abbreviations

HIV-1: human immunodeficiency virus type I
AIDS: acquired immunodeficiency syndrome
Env: envelope glycoproteins
smFRET: single-molecule fluorescence resonance energy transfer
bNAbs: broadly neutralizing antibodies
